# Bio-Guided Isolation of Antimalarial Metabolites from the Coculture of Two Red Sea Sponge-Derived *Actinokineospora* and *Rhodococcus* spp.

**DOI:** 10.3390/md19020109

**Published:** 2021-02-12

**Authors:** Hani A. Alhadrami, Bathini Thissera, Marwa H. A. Hassan, Fathy A. Behery, Che Julius Ngwa, Hossam M. Hassan, Gabriele Pradel, Usama Ramadan Abdelmohsen, Mostafa E. Rateb

**Affiliations:** 1 Department of Medical Laboratory Technology, Faculty of Applied Medical Sciences, King Abdulaziz University, P.O. BOX 80402, Jeddah 21589, Saudi Arabia; hanialhadrami@kau.edu.sa; 2 Molecular Diagnostic Laboratory, King Abdulaziz University Hospital, King Abdulaziz University, P.O. BOX 80402, Jeddah 21589, Saudi Arabia; 3 School of Computing, Engineering & Physical Sciences, University of the West of Scotland, Paisley PA1 2BE, UK; bathini.thissera@uws.ac.uk; 4 Department of Pharmacognosy, Faculty of Pharmacy, Beni-Suef University, Beni-Suef 62514, Egypt; mh_elsefy@yahoo.com (M.H.A.H.); abuh20050@yahoo.com (H.M.H.); 5 Department of Pharmacognosy, Faculty of Pharmacy, Mansoura University, Mansoura 35516, Egypt; fathy.behery@riyadh.edu.sa; 6 Department of Pharmacy, College of Pharmacy, Riyadh Elm University, Riyadh 11681, Saudi Arabia; 7 Division of Cellular and Applied Infection Biology, Institute of Zoology, RWTH Aachen University, 52074 Aachen, Germany; ngwa.che@bio2.rwth-aachen.de (C.J.N.); pradel@bio2.rwth-aachen.de (G.P.); 8 Department of Pharmacognosy, Faculty of Pharmacy, Nahda University, Beni-Suef 62514, Egypt; 9 Department of Pharmacognosy, Faculty of Pharmacy, Minia University, Minia 61519, Egypt; 10 Department of Pharmacognosy, Faculty of Pharmacy, Deraya University, New Minia 61111, Egypt

**Keywords:** *Actinokineospora*, *Rhodococcus*, co-culture, metabolomics, antimalarial, docking

## Abstract

Coculture is a productive technique to trigger microbes’ biosynthetic capacity by mimicking the natural habitats’ features principally by competition for food and space and interspecies cross-talks. Mixed cultivation of two Red Sea-derived actinobacteria, *Actinokineospora spheciospongiae* strain EG49 and *Rhodococcus* sp. UR59, resulted in the induction of several non-traced metabolites in their axenic cultures, which were detected using LC–HRMS metabolomics analysis. Antimalarial guided isolation of the cocultured fermentation led to the isolation of the angucyclines actinosporins E (**1**), H (**2**), G (**3**), tetragulol (**5**) and the anthraquinone capillasterquinone B (**6**), which were not reported under axenic conditions. Interestingly, actinosporins were previously induced when the axenic culture of the *Actinokineospora spheciospongiae* strain EG49 was treated with signalling molecule *N*-acetyl-d-glucosamine (GluNAc); this finding confirmed the effectiveness of coculture in the discovery of microbial metabolites yet to be discovered in the axenic fermentation with the potential that could be comparable to adding chemical signalling molecules in the fermentation flask. The isolated angucycline and anthraquinone compounds exhibited in vitro antimalarial activity and good biding affinity against lysyl-tRNA synthetase (PfKRS1), highlighting their potential developability as new antimalarial structural motif.

## 1. Introduction

Exploring microbial forms of communication and utilising them in the production of secondary metabolites is of benefit in the process of natural products drug discovery [1]. Thus far, microbial secondary metabolites remain the major source for antimicrobial agents [2,3,4]. However, gene sequencing of many microbial genome showed that several species, mainly filamentous bacteria and fungi, apply a considerable part of their genes for secondary metabolism (10–15%) [5,6]. Remarkably, most microorganism genes are silent and have no role during laboratory cultivation [7]. Robert Koch used cultures for only one species of microorganism “axenic growth” to provide an apparent elucidation for this phenomenon of silent genes [8]. Microorganism culture in laboratory included macro- and micro-nutrients, constant temperature, adjusted pH, high water activity, and no contact with other world microbes, and thus a significant part of microorganisms’ secondary metabolites, mainly those responsible for interaction, communication, or involving in fights with other species, are not found in microbial metabolites. Therefore, the new approach of co-cultivation provides a massive chance to motivate the silent genes and increase the opportunity to discover cryptic bioactive metabolites [1]. The fortune of “uncultivable” diversity is represented as the “microbial dark matter”, the part of microorganisms that was unable to be cultivated in the laboratory until now [1]. The first reported mixed culture was in 1918; it was a coculture of *Escherichia coli* and *Bacillus paratyphosus* [9]. Up until now, natural product discovery, biotechnology, and microbiology scientists work on the discovery of coculture or mixed culture experiments to study the difference in the secondary metabolites produced during these trials compared to “axenic growth” [1]. From the examples for coculture and secondary metabolites production, mixed culture of *Acremonium* sp. and *Mycogonerosea* that produced new lipoaminopeptides, the acremostatins A-C [10]. Coculture of the marine-derived fungi *Aspergillus fumigatus* together with two desert bacterial isolates yielded new compounds, namely, luteoride D and pseurotin G [11]. Furthermore, a new N-methoxypyridone was discovered from a mixed fermentation of two endophytic fungi *Camporesia sambuci* and *Epicoccum sorghinum* isolated from the fruit of *Rhodomyrtus tomentosa* plant, collected on the Big Island in Hawaii [12]. New antifungal pulicatin derivatives H and I were induced following coculturing of plant-derived bacterium *Pantoea agglomerans* and the fungus *Penicillium citrinum* [13]. All these examples exemplify that co-cultivation of microorganisms induces new secondary metabolites that can be recommended as an appropriate way to produce diverse bioactive microbial metabolites.

Malaria was identified as a lethal disease caused by *Plasmodium* parasites, which infect humans through the malaria vector Anopheles mosquitoes. To date, five species of parasites have been identified as causatives of malaria in humans; two of them cause serious infections—*P. falciparum* and *P. vivax*. Studying the malaria cases worldwide revealed that 29 countries accounted for 95% of malaria cases. The majority of cases (82%) and deaths (94%) were reported in the WHO African region, followed by the WHO South-East Asia region (10% cases and 3% deaths) (https://www.who.int/publications/i/item/9789240015791, accessed on 30 January 2021). Malaria management and suppression require a complicated method. Up until now, two important antimalarial drugs are used to control infection. These two bitter principle drugs are derived from plants: artemisinin obtained from *Artemisia annua* L. (4th century, China), and quinine alkaloid obtained from *Cinchona* sp. (17th century, South America) [14]. The WHO recommends artemisinin combination therapy (ACT) as the first treatment plan in most malarial cases. However, in 2009, resistance to artemisinin combination therapy was reported. The emerging of drug resistance led to increased malaria cases and an increase in mortality [15]. Thus, the WHO endorsed using a combination of two drugs that work in different mechanisms to control drug resistance. The latest reports from Southeast Asia and India [16] showed the limitation of disease resistance to combination of artemisinin and other drugs as mefloquine and piperaquine [17]. Lacking effective new generation of medicines against malarial invasion, the number of new cases and deaths may rise. Thus, developing antimalarial therapeutics is important to save a large number of lives.

In this work, we discuss the application of co-cultivation of two actinobacteria: *Actinokineospora spheciospongiae* strain EG49 and *Rhodococcus* sp. UR59 recovered from Red Sea sponges as a strategy to stimulate silent genes and discover cryptic secondary metabolites within both strains. Additionally, antimalarial-guided fractionation of the bacterial coculture extract led to the isolation and characterisation of a few active metabolites against *P. falciparum*. A potential antimalarial target is proposed on the basis of molecular docking experiments against a number of reported targets.

## 2. Results and Discussions

### 2.1. Identification of Red Sea Sponge-Associated Actinobacteria

**Figure 1:**
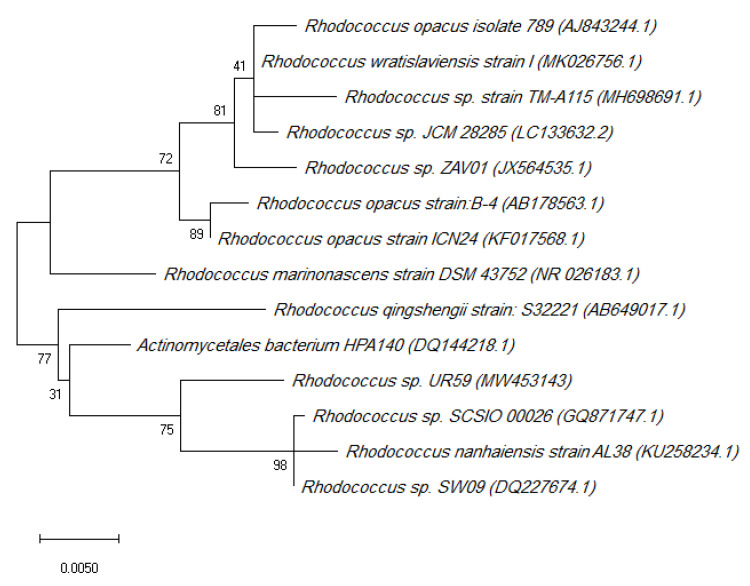
Phylogenetic tree of the *Rhodococcus* sp. UR59 isolate and the closest relatives in terms of the 16S rRNA gene marker. The accession numbers are indicated in brackets.

Two Red sea sponge-associated actinobacteria were isolated and taxonomically identified. *Actinokineospora spheciospongiae* strain EG49 was previously characterised [18,19]. The other actinobacterial strain was taxonomically identified as *Rhodococcus* sp. UR59, according to its morphology and its 16S rRNA genome sequence and phylogenetic analyses (Figure 1). 

### 2.2. Metabolomics Analysis of the Coculture Extract of Actinokineospora spheciospongiae Strain EG49 and Rhodococcus sp. UR59 Using LC–HRMS

The analysis of the metabolomics data (Table 1) revealed 34 microbial secondary metabolites, of which 9 were detected from *Actinokineospora spheciospongiae* strain EG49 and the rest from *Rhodococcus* sp. UR59. Additionally, the analysis revealed the presence of diverse microbial chemical classes, namely, 10 angucyclines, 7 peptides, 3 macrolides, 3 anthraquinones, 2 polyenes, 2 polyethers, 2 phenolics, and 1 glycolipid. The predicted formula C_16_H_18_N_2_O_4_ was annotated as mitomycin-K [20,21], whereas C_18_H_14_O_6_ was dereplicated as fluostatin-B, an inhibitor of dipeptidyl peptidase III that was previously isolated from *Streptomyces* sp. TA-3391 [22]. Moreover, the predicted formulas C_32_H_33_O_15_ and C_31_H_33_O_13_ were dereplicated as actinosporin A and C, respectively, which were discovered from the culture of *Actinokineospora spheciospongiae* strain EG49 [23,24]. The formula C_21_H_18_O_8_ was dereplicated as daunomycinone, which was reported from *Streptomyces coeruleorubid* [25]. The formulas C_26_H_25_O_11_ and C_25_H_24_O_8_ were dereplicated as atramycin A and B, respectively. These isotetracenone metabolites were discovered from *Streptomyces atratus* BY90 [26]. Additionally, the suggested molecular formula C_18_H_12_O_5_ was dereplicated as lagumycin B, which was previously isolated from *Micromonospora* sp. [27], while the formula C_16_H_12_O_5_ was dereplicated as the isoflavonoid kakkatin that was reported from the soil-derived *Streptomyces* strain YIM GS3536. Moreover, it was discovered in another terrestrial *Streptomyces* sp. GW39/1530 [28,29]. Furthermore, the molecular formula C_9_H_9_NO_3_ was dereplicated as erbstatin, a simple dehydrotyrosine derivative isolated from *Streptomyces amnkusaensis* [30,31]. Additionally, the molecular formula C_36_H_48_N_2_O_8_ was dereplicated as ansatrienin A, previously detected in *Streptomyces collinus* [32]. Moreover, the formulas C_25_H_47_N_5_O_4_, C_26_H_49_N_5_O_4_, and C_28_H_53_N_5_O_4_ were dereplicated as cyclic tetrapeptides rhodopeptin C1, C2, and B5, respectively, which were formerly reported in *Rhodococcus* sp. [33,34]. The formula C_32_H_48_N_6_O_9_ was dereplicated as the peptide actinoramide B, which was detected in a marine bacterium highly corelated to the genus *Streptomyces* [35]. Likewise, the formula C_17_H_26_O_4_ was dereplicated as cineromycin-B antibiotic that showed significant MRSA inhibition, which was isolated from the actinomycetales strain INA 2770 [36]. The formula C_19_H_27_N_5_O_7_ was annotated as heterobactin B, a siderophore discovered from *Rhodococcus erythropolis* IGTS8 [37], while the formula C_26_H_39_NO_5_ was dereplicated as piericidin-F, which was reported from *Streptomyces* sp. CHQ-64 [38]. Additionally, the formula C_27_H_39_NO_7_ was annotated as migrastatin, which was reported as a tumour cell migration inhibitor and isolated from *Streptomyces* sp. MK929-43F1 [39]. Moreover, the formula C_24_H_46_N_6_O_8_ was dereplicated as proferrioxamine-A1, a siderophore isolated from *Streptomyces xinghaiensis* NRRL B-24674T [40]. Furthermore, the formula C_23_H_38_O_5_ was dereplicated as the 16-membered lactone protylonolide, which was identified as the metabolite of mycaminose idiotroph that has been obtained from *Streptomyces fradiae* KA-427 [41]. Moreover, the formula C_37_H_62_O_11_ was dereplicated as the polyether 26-deoxylaidlomycin isolated from *Streptoverticillium olivoreticuli* IMET 43,782 [42], while the suggested formula C_35_H_58_O_10_ was dereplicated as macrolide kaimonolide B, which was discovered in *Streptomyces* sp. no. 4155 and shown to significantly inhibit plant growth [43]. Furthermore, the formula C_25_H_44_O_7_ was dereplicated as 8,15-dideoxylankanolide, which was reported in *Streptomyces rochei* 7434AN4 [44]. The molecular formula C_34_H_60_O_10_ was identified as the polyether antibiotic ferensimycin-A, previously discovered in *Streptomyces* sp. no. 5057 [45]. Likewise, the formula C_26_H_46_N_6_O_5_ was identified as the cytotoxic peptide lucentamycin C, which was reported from a marine-derived actinomycete *Nocardiopsis lucentensis* CNR-712 [46]. Finally, the formula C_50_H_92_O_14_ was dereplicated as glucolipsin-A, a glucokinase activator that has been isolated from *Streptomyces puvpuvogenisclevoticus* [47]. 

It is worth noting that the compounds listed in Table 1 were traced in the LC–HRESIMS analysis of the coculture extract. The producing strain for each compound was predicted on the basis of literature. However, mitomycin-K, 8,15-dideoxylankanolide, piericidin-F, migrastatin, kaimonolide B, rhodopeptin C1, rhodopeptin C2, and rhodopeptin B5 were also traced in the axenic culture of *Rhodococcus* sp. UR59. Additionally, actinosporins A and C, and UK-2B were also traced in the axenic culture of *Actinokineospora spheciospongiae* strain EG49. All other reported metabolites in Table 1 were not traced in the axenic cultures and were induced during the coculture fermentation. 

### 2.3. Identification of the Isolated Compounds (***1***–***8***)

**Figure 2:**
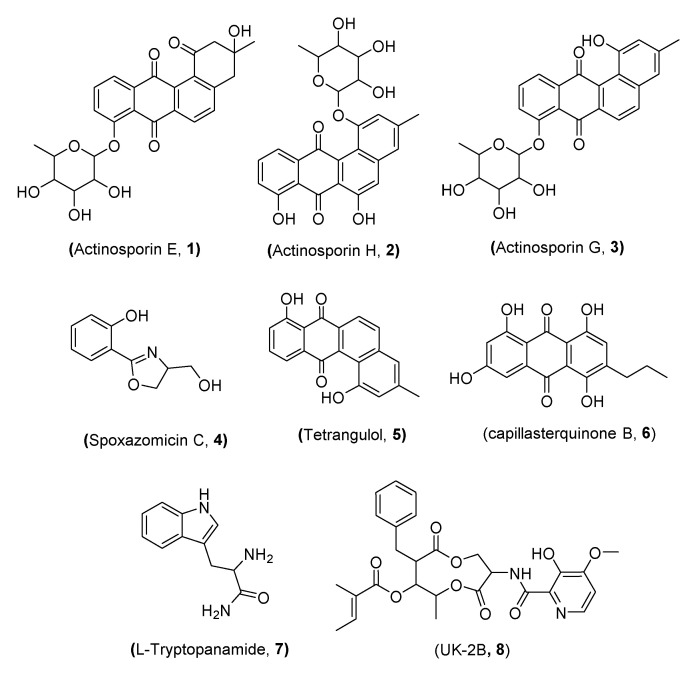
Compounds isolated from the coculture of *Actinokineospora spheciospongiae* strain EG49 and *Rhodococcus* sp. UR59.

Chemical structures of the purified metabolites **1–8** from the coculture were assigned on the basis of comparing the LC–HRESIMS analysis, 1D and 2D NMR spectral data, and optical rotation measurements to the published literature (Figure 2). Accordingly, compounds **1–3** have been previously isolated from *Actinokineospora spheciospongiae* strain EG49 and identified as the angucyclinone antibiotics actinosporin E, H, and G, respectively, through the activation of their cryptic gene cluster by N-acetylglucosamine [50]. Compound **4** was assigned as spoxazomicin C of the pyochelin family of antibiotics, which was previously isolated from the culture broth of the endophyte *Streptosporangium oxazolinicum* K07-0460T [51]. In contrast, compound **5** was previously identified as the angucyclinone antibiotic tetrangulol, which was previously isolated from *Streptomyces rimosus* [58] and recently from *Amycolatopsis* sp. HCa1 [59]. Compound **6** was previously discovered as capillasterquinone B, an anthraquinone that was isolated from the crinoid *Capillaster multiradiatus* [57]. Moreover, compound **7** was identified as L-tryptophanamide. We propose it as an artefact as it was not traced in the LC–MS analysis of either the axenic or the coculture extracts, and thus it was probably generated during the fractionation and purification process. Finally, compound **8** was isolated from *Streptomyces* sp. 517-02 [57] and identified as UK-2B, an antifungal antibiotic with similarity in structure to antimycin A [60].

The same *Actinokineospora spheciospongiae* strain EG49 was subjected to N-acetyl-D-glucosamine (GluNAc)-mediated silent gene activation to produce new actinosporins E–H, the same actinosporins E (**1**), G (**3**), and H (**2**) discussed here under coculture and aglycone angucycline tetrangulol (**5**), which was not reported from the axenic culture treated with GluNAc [50]. The amino sugar GluNAc is a signalling molecule that can induce microbial secondary metabolism. It is present as a cell wall component in peptidoglycan or chitin in the bacterial or fungal cell wall, respectively [60,61]. Having observed a similar induction when *Actinokineospora spheciospongiae* strain EG49 was cocultured with *Rhodococcus* sp. UR59, we can assume that *Rhodococcus* sp. UR59 directed the biosynthesis of actinosporins in a similar way to GluNAc. This could be as exudation of GluNAc by one of the species into the coculture environment to trigger antibiotic production more likely from *Rhodococcus* sp. UR59 as defence molecules. The studies further support this and demonstrated that GluNAc is secreted by bacteria under malnourished conditions to signal antibiotic production against opposite competitors in the vicinity [62]. However, this requires further studies on the coculture medium to identify excreted GluNAc or compounds with similar signalling function.

### 2.4. Antimalarial Screening

Angucyclines are microbial secondary metabolites known as promising antimicrobial, anticancer, and antimalarial agents [63,64,65]. The core structure of angucyclines is characterised by a benz[α]anthracene ring, an angular tetracycline ring system [60]. The reported angucyclines can be categorised as aglycones such as saccharosporones A, B, and C [60], and glycosylated angucyclines such as pseudonocardones A−C [63] and urdamycinone E, urdamycinone G, and dehydroxyaquayamycin isolated from fungal and bacterial strains [62]. However, different antimalarial activity profiles between aglycones and glycosylated angucyclines have not been explained. 

The potential antiparasitic effectiveness of the angucycline scaffold and the promising antimalarial effect exhibited by the total extract of the coculture of *Actinokineospora spheciospongiae* strain EG49 and *Rhodococcus* sp. UR59 (IC_50_ value of 0.13 µg/mL, Table 2) when screened against *Plasmodium falciparum* have encouraged us to perform large-scale coculture fermentation. Large-scale fermentation followed by liquid–liquid fractionation and HPLC purification of the active sub-fraction led to the isolation of eight metabolites. The antimalarial screening of the isolated compounds indicated that the angucycline glycosides **1**–**3** and aglycone **5** and the anthraquinone **6** exhibited antimalarial effect with IC_50_ values in the range of 9–13.5 μg/mL in comparison to the IC_50_ value of the positive control chloroquine (0.022 μg/mL). The activity of the compounds **1**–**3**, **5**, and **6** was further studied by docking against a few known drug targets to suggest these compounds as potential leads to be developed for enhanced activity. It worth noting that the isolated molecules did not show the expected antimalarial activity, which could be attributed to either the synergistic effect of microbial metabolites in the coculture extract or the presence of minor molecules that were too scarce to be isolated even after large-scale fermentation. 

### 2.5. Docking Analysis

**Figure 3:**
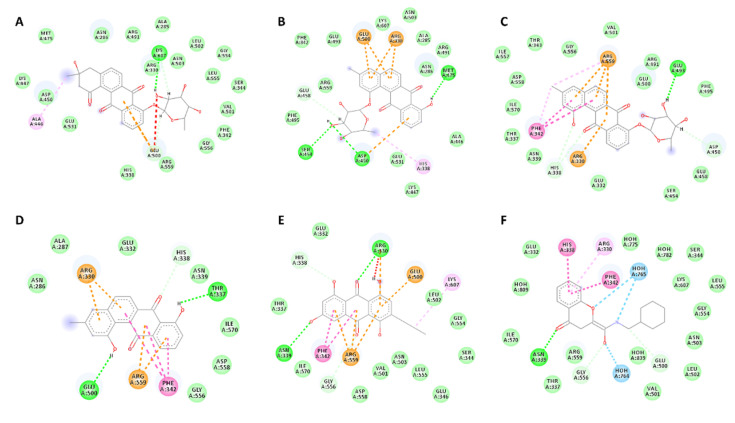
Binding modes of compounds **1**–**3**, **5**, and **6** ((**A**–**E**) respectively) together with the co-crystalised ligand (**F**) inside PfKRS’s active site. Dashed lines indicate interactions between each ligand and the active site’s amino acid residues. Green colour indicates H-bonding; orange colour indicates π–anion or π–cation interactions; pink colour indicates hydrophobic interactions.

Compounds (**1**–**3**, **5**, **6**) that showed inhibitory activity against *P. falciparum* were subjected to molecular docking experiments against a number of reported malaria targets, e.g., NADH:ubiquinone oxidoreductase (PDB: 5JWA), Kelch protein (PDB: 4YY8), *P. falciparum* protein kinase (PDB: 1V0P), NADH dehydrogenase 2 (PDB:4PD4), and lysyl-tRNA synthetase (PDB:6AGT). They achieved the best scores (binding energy −8.5 to −9.1 kcal/mol) against the later target, lysyl-tRNA synthetase (PfKRS1). Moreover, they exhibited binding mode inside the active site compared to the co-crystalised ligand [66]. As shown in Table 3 and Figure 3, these compounds exhibited multiple interactions with several amino acids inside the enzyme’s active site, where ARG-330, HIS-338, GLU-500, ARG-559, and PHE-342 were the most common interacting ones. Hence, this attractive scaffold can be utilised in the future design of antimalarial therapeutics targeting PfKRS1 (Table 3). Antimalarial effect of the bacterial coculture derived metabolites.

## 3. Materials and Methods

### 3.1. General Experimental

Extract purification was conducted by preparative Agilent 1100 series HPLC equipped with gradient pump and DAD using a reversed-phase Sunfire (C18, 5 µm, 10 × 250 mm, serial no. 226130200125). All 1D and 2D NMR spectral data were acquired using a JEOL ECZ-R500 NMR spectrometer equipped with a Royal 5 mm combined broadband and inverse probe. Thermo LTQ Orbitrap coupled to an HPLC system was utilised to acquire HRESIMS data using capillary temperature of 260 °C, capillary voltage of 45 V, sheath gas flow rate of 40–50 arbitrary units, auxiliary gas flow rate of 10–20 arbitrary units, spray voltage of 4.5 kV, and mass range of 100–2000 amu (maximal resolution of 60,000). Optical rotations and UV spectra acquisition were acquired using a Perkin-Elmer 343 polarimeter and Perkin-Elmer Lambda2 UV–VIS spectrometer, respectively. 

### 3.2. Actinomycetes Isolation

*Callyspongia* sp. was collected from Hurghada (Red Sea, Egypt) at a depth of 5 m and latitude 27°17′01.0′′ N and longitude 33°46′21.0′′ E. The sponge specimen was identified by Prof. El-Sayd Abed El-Aziz (Department of Invertebrates Lab., National Institute of Oceanography and Fisheries, Egypt). The sponge was transported in a plastic bag in seawater to the laboratory and washed thoroughly with sterile seawater. The surface sterilised specimen was cut into pieces of ≈1 cm^3^, followed by vigorous homogenising with 10 volumes of sterile seawater in a pre-sterilised mortar. Serially diluted supernatant (10^−1^, 10^−2^, 10^−3^) was subsequently plated on to the sterile agar plates. For the isolation of different actinomycetes, we used M1, ISP2, and marine agar (MA) media were used [18]. The isolation of slow-growing actinomycetes was performed by supplementing all media with filtered 25 µg/mL nalidixic acid, 25 µg/mL nystatin, and 100 µg/mL cycloheximide. The inoculated plates were stored in an incubator for 6–8 weeks at 30 °C. Subculturing of distinct colony morphotypes resulted in pure strains. *Rhodococcus* sp. UR59 was cultured on ISP2 medium and preserved in 20% glycerol at −80 °C. On the other hand, *Actinokineospora spheciospongiae* strain EG49 was previously recovered and identified from the Red Sea sponge *Spheciospongia vagabunda* [18]. 

### 3.3. Molecular Identification and Phylogenetic Analysis

With reference to Hentschel et al., we carried out 16S rRNA gene amplification, cloning, and sequencing using 27F and 1492RRNA as universal primers [18]. By using the Pintail programme, we identified chimeric sequences [67]. The sequence’s genus level affiliation was validated using the Project Classifier of the Ribosomal Database. All the sequences were classified at the genus level by the RDP Classifier (g 16srrna, f allran) and confirmed with the SILVA Incremental Aligner (SINA) [68]. Using the SINA Web Aligner, an alignment was determined again (variability profile: bacteria). The Gap-only position with trimALL was eliminated (-noallgaps). The best fitting model was initially calculated for phylogenetic tree construction with the Model Generator. To produce the phylogenetic tree, we applied RAxML (-f a-m GTRGAMMA-x 12345-p 12345 -# 1000) and the estimated model with 1000 bootstrap resamples. With Interactive Tree of Life (ITOL) [69], visualisation was achieved. The BLAST with the accession number MW453143 was deposited at Genebank.

### 3.4. Co-Cultivation and Extract Preparation 

*Rhodococcus* sp. UR59 and *Actinokineospora spheciospongiae* strain EG49 were cultivated on liquid media M1 and ISP2 as axenic and cocultures. A total of 20 mL of 3-day-old culture of *Rhodococcus* sp. was used for large scale fermentation. *Rhodococcus* sp. UR59 was transferred to 20 × 2 L Erlenmeyer flasks containing 1 L of ISP2 medium pre-inoculated with 20 mL of 4-day-old *Actinokineospora spheciospongiae* strain EG49 and left for 7 days at 25 °C and 180 rpm in a shaker incubator. After fermentation, the culture was filtered, and the supernatant was extracted twice with ethyl acetate (1.5 L each) followed by evaporation under vacuum to provide the ethyl acetate extract (850 mg).

### 3.5. Metabolic Profiling

For mass spectrometry analysis, the dry ethyl acetate extracts from different microbial and coculture samples were dissolved in MeOH at 1 mg/mL and subjected to metabolic analysis using LC–HRESIMS according to Abdelmohsen et al. [23]. An Acquity UPLC system coupled to a Synapt G2 HDMS qTOF hybrid mass spectrometer (Waters, Milford, CT, USA) was used to acquire the HRMS data using capillary temperature at 320 °C, spray voltage at 4.5 kV, and mass range of *m/z* 150–1500; both positive and negative ESI modes were applied. The MS was processed using MZmine 2.20 on the basis of the defined parameters [23]. The chromatogram builder and chromatogram deconvolution were detected and followed by mass ion peaks. The isotopes were differentiated by grouper isotopic peaks and the missing peaks were depicted using the gap-filling peak finder. Then, molecular formula prediction and peak identification were conducted from the processed positive and negative ionisation mode datasets. Finally, the peaks were dereplicated against the Dictionary of Natural Products (DNP) database. 

### 3.6. Metabolites Isolation 

The crude co-fermentation ethyl acetate (EtOAc) (850 mg) was chromatographed on Sephadex LH-20 (32–64 µm, 100 × 25 mm) column using an 80:20 MeOH/H_2_O eluent in order to obtain 5 fractions (Fr.1–Fr.6). The third bioactive fraction (300 mg) was then chromatographed using silica gel column with a gradient elution starting at DCM/EtOAc (100:0 to 0:100) then 100% MeOH to obtain 8 sub-fractions. The active subfractions 4 and 5 were combined (85 mg) and further subjected to semi preparative HPLC purification (Sunfire, C18, 5 µm, 10 × 250 mm) with a gradient of 20%–100% CH_3_CN in H_2_O over 30 min and 10 min at 100% CH_3_CN at 1.5 mL/min flow rate to yield compound **7** (t_R_ 9.6 min, 7.5 mg), **2** (t_R_ 10.7 min, 4.5 mg), **3** (t_R_ 11.2 min, 2.5 mg), **4** (t_R_ 15.2 min, 2.8 mg), **5** (t_R_ 18.3 min, 2.1 mg), **1** (t_R_ 24.6 min, 3.2 mg), **6** (t_R_ 27.2 min, 3.8 mg), and **8** (t_R_ 31.3 min, 1.5 mg). 

### 3.7. Antimalarial Screening

The Malstat assay was used as mentioned earlier to assess the compounds’ antimalarial effect [70,71]. The compounds were dissolved in DMSO (Sigma Aldrich, Taufkirchen, Germany) at concentrations ranging from 50 μg/mL to 0.4 μg/mL, and synchronised *P. falciparum* 3D7 ring stage cultures were placed in duplicate at a parasite level of 1% in 96-well plates (200 μL/well). Chloroquine (CQ; Sigma Aldrich, Taufkirchen, Germany) was used as a positive control. The *P. falciparum* 3D7 parasite was cultured with the compounds at 37 °C in 5% O_2_, 5% CO_2_, and 90% N_2_ for 72 h. After this, 20 µL was transferred to 100 µL of the Malstat reagent (0.1% Triton X-100, 1 g of L-lactate, 0.33 g Tris, and 33 mg of APAD (3-acetylpyridine adenine dinucleotide; Taufkirchen, Germany)) dissolved in 100 mL of distilled water (pH 9.0) in a 96-well microtiter plate. The plasmodial lactate dehydrogenase (LDH) activity was then evaluated by adding to the Malstat reaction 20 μL of a 1:1 mixture of diaphorase (1 mg/mL) and nitro blue tetrazolium (NBT). The optical densities were estimated at 630 nM, and the IC_50_ values were determined using the GraphPad Prism software version 5 from variable-slope sigmoidal dose–response curves (GraphPad Software Inc., La Jolla, CA, USA).

### 3.8. Molecular Docking

Docking analysis was carried out using the Discovery Studio 2.5 software (Accelrys Inc., San Diego, CA, USA). Completely automatic docking tool using “Dock ligands (CDOCKER)” procedure operating on Intel Core i32370 CPU @ 2.4 GHz 2.4 GHz, RAM Memory 2 GB under the Windows 10.0 system. Furthermore, these docked compounds were assembled using a software Chem 3D ultra 12.0 (Cambridge Soft Corporation, USA (2010)), and then sent to the Discovery Studio 2.5 software. From this, an automatic protein formulation procedure was conducted through the MMFF94 forcefield with the binding site sphere recognised by the software. The receptor was recorded as “input receptor molecule” in the CDOCKER protocol explorer. Establishing this, the test compounds were subjected to force fields to obtain the minimum energy structure. These poses were ranked and studied thoroughly, showing the best ligand–HDAC interactions from the calculations and 2D and 3D examinations [72,73].

## 4. Conclusions

Microbial coculture continues to prove its efficiency in triggering the production of cryptic microbial secondary metabolites. Mixed cultivation of two Red Sea-derived actinobacteria, namely, *Actinokineospora spheciospongiae* strain EG49 and *Rhodococcus* sp. UR59, resulted in the induction of several non-traced metabolites in their axenic cultures. Interestingly, actinosporins E–H were reported to be induced when the axenic culture of the *Actinokineospora spheciospongiae* strain EG49 was treated with the signalling molecule GluNAc. Such induction was comparable to that made by the *Rhodococcus* sp. UR59 in the coculture environment, providing the effectiveness of co-cultivation in the discovery of microbial metabolites yet to be discovered in the axenic fermentation with the potential that could be comparable to adding signalling molecules in the fermentation flask. Additionally, the induced actinosporins exhibited a promising antimalarial effect that is likely to be through the inhibition of *P. falciparum* lysyl-tRNA synthetase, which requires further investigation as an interesting structural motif for the development of new antimalarial therapeutics.

## Figures and Tables

**Table 1:** Metabolomics analysis of the coculture extract of *Actinokineospora spheciospongiae* strain EG49 and *Rhodococcus* sp. UR59.

Rt (min)	*m*/*z* [M − H]^−^	*m*/*z* [M + H]^+^	Molecular Formula	Tentative Identification	Strain EG49	Strain UR59	Coculture	Bioactivity	Ref.
2.47		303.1341	C_16_H_18_N_2_O_4_	Mitomycin-K	-	+	+	antitumor	[20]
2.91		327.0866	C_18_H_14_O_6_	Fluostatin-B	-	-	+	antinociceptive	[22]
2.94	657.1821		C_32_H_33_O_15_	Actinosporin A	+	-	+	anti-trypanosomal	[23]
2.96		613.1926	C_31_H_33_O_13_	Actinosporin C	+	-	+	antioxidant	[24]
2.99		399.1075	C_21_H_18_O_8_	Daunomycinone	-	-	+	-	[48]
3.04		599.2125	C_31_H_34_O_12_	Actinosporin F	-	-	+	-	[49]
3.08		469.1492	C_25_H_24_O_9_	Actinosporin E	-	-	+	-	[49]
3.11		467.1336	C_25_H_22_O_9_	Actinosporin H	-	-	+	-	[49]
3.15	513.1399		C_26_H_25_O_11_	Atramycin A	-	-	+	antitumor	[26]
3.25		451.1389	C_25_H_22_O_8_	Actinosporin G	-	-	+	-	[49]
3.30		309.0757	C_18_H_12_O_5_	Lagumycin B	-	-	+	anticancer	[27]
3.76	178.0499		C_9_H_9_NO_3_	Erbstatin	-	-	+	anticancer	[30]
3.81	635.3315		C_36_H_48_N_2_O_8_	Ansatrienin A	-	-	+	antifungal	[32]
3.93	192.0655		C_10_H_11_NO_3_	Spoxazomicin C	-	-	+	anti-trypanosomal	[50]
4.12		661.3568	C_32_H_48_N_6_O_9_	Actinoramide B	-	-	+	antimalarial	[51]
4.17	293.1749		C_17_H_26_O_4_	Cineromycin-B	-	-	+	antibacterial	[52]
4.54		438.1974	C_19_H_27_N_5_O_7_	Heterobactin B	-	-	+	siderophore	[37]
6.41	451.1391		C_25_H_24_O_8_	Atramycin B	-	-	+	antitumor	[26]
6.34	444.2744		C_26_H_39_NO_5_	Piericidin-F	-	+	+	anticancer	[38]
6.91	488.2649		C_27_H_39_NO_7_	Migrastatin	-	+	+	anticancer	[53]
7.24		547.3455	C_24_H_46_N_6_O_8_	Proferrioxamine-A1	-	-	+	siderophore	[40]
7.40	393.2640		C_23_H_38_O_5_	Protylonolide	-	-	+	antibiotic	[41]
7.58		683.4347	C_37_H_62_O_11_	26-Deoxylaidlomycin	-	-	+	antibacterial	[54]
7.76		482.3687	C_25_H_47_N_5_O_4_	Rhodopeptin C1	-	+	+	Antifungal	[33]
8.15		639.4084	C_35_H_58_O_10_	Kaimonolide B	-	+	+	plant growth inhibitor	[43]
9.09		496.3846	C_26_H_49_N_5_O_4_	Rhodopeptin C2	-	+	+	antifungal	[34]
9.14		524.4156	C_28_H_53_N_5_O_4_	Rhodopeptin B5	-	+	+	antifungal	[34]
9.80		315.0865	C_17_H_14_O_6_	Capillasterquinone B	-	-	+	NO production inhibitor	[55]
9.81		305.0810	C_19_H_12_O_4_	Tetrangulol	-	-	+	antibiotic	[56]
9.98		457.3141	C_25_H_44_O_7_	8,15-Dideoxylankanolide	-	+	+	-	[44]
10.22		527.2022	C_27_H_31_N_2_O_9_	UK-2B	+	-	+	antifungal	[57]
11.01		629.4242	C_34_H_60_O_10_	Ferensimycin-A	-	-	+	antibiotic	[45]
11.23		523.3601	C_26_H_46_N_6_O_5_	Lucentamycin C	-	-	+	anticancer	[46]
11.28		917.6546	C_50_H_92_O_14_	Glucolipsin-A	-	-	+	glucokinase activator	[47]

**Table 2:** Antimalarial effect of the bacterial coculture derived metabolites.

Compound	IC_50_ Values (µg/mL) ^1^
Coculture extract	0.13
**1**	12.6
**2**	13.6
**3**	11.2
**4**	>50
**5**	9.7
**6**	9.2
**7**	>50
**8**	>50
Chloroquine	0.022

^1^ Average of two independent runs.

**Table 3:** Binding scores and interacting amino acid residues with compounds **1**–**3**, **5**, and **6** inside the lysyl-tRNA synthetase (PfKRS1)’s active site.

Compound	Binding Energy (kcal/mol)	H-Bonding	Hydrophobic Interactions
**1**	−8.9	ARG-330	ALA-446, GLU-500, LYS-607
**2**	−8.3	ASP-450, SER-454, MET-475	ARG-330, HIS-338, ASP-450, GLU-458, GLU-500
**3**	−10.3	GLU-493	ARG-330, PHE-342, ASP-450, ARG-559
**5**	−9.1	GLU-500, THR-337	ARG-330, HIS-338, PHE-342, ARG-559
**6**	−9.0	ARG-330, ASN-339	ARG-330, HIS-338, PHE-342, GLU-500, ARG-559, LYS-607
Co-crystalised ligand	−9.5	ASN-339, GLY-556	ARG-330, HIS-338, PHE-342

## Data Availability

No supplementary data available with this article.
